# MOSAL: software tools for multiobjective sequence alignment

**DOI:** 10.1186/1751-0473-9-2

**Published:** 2014-01-08

**Authors:** Luís Paquete, Pedro Matias, Maryam Abbasi, Miguel Pinheiro

**Affiliations:** 1 CISUC, Department of Informatics Engineering, University of Coimbra, Polo II, 3030-290 Coimbra, Portugal; 2 School of Medicine, University of St. Andrews, North Haugh, KY16 9TF St. Andrews, UK

**Keywords:** Pairwise sequence alignment, Multiobjective optimization, Dynamic programming

## Abstract

Multiobjective sequence alignment brings the advantage of providing a set of alignments that represent the trade-off between performing insertion/deletions and matching symbols from both sequences. Each of these alignments provide a potential explanation of the relationship between the sequences. We introduce MOSAL, a software tool that provides an open-source implementation and an on-line application for multiobjective pairwise sequence alignment.

## Background

Sequence alignment is in the core of many bioinformatics applications. It aims to identify regions of similarity in sequences of biological data, such as nucleotide and amino acid residues. The procedure consists of inserting gaps between the residues so that similar symbols from several sequences become aligned. For two sequences, dynamic programming algorithms can compute the optimal alignment in an efficient manner [1]. However, for very large DNA or protein databases, heuristic approaches like FASTA and BLAST have been used [2,3]. See [4] for an extensive review from a computational point of view.

Any of these approaches rely on the a priori definition of coefficients that are assigned to the components of the score function. These weights are usually defined by default in most of the software packages for sequence alignment and are usually not modified by the practitioner. However, there is a considerable disagreement about how to weight each coefficient. A small change in the weights can lead to a completely different alignment.

One way of overcoming the problem of setting weights is to consider a multiobjective formulation, where the practitioner is provided a set of optimal alignments representing the trade-off between components of the score function, for instance, substitution score given by a substitution matrix and the number of gaps; in this case, an alignment is optimal if there is no other alignment with better substitution score value and lesser number of gaps. Usually, there is not only one optimal alignment but several for which this notion of optimality holds; such set of all optimal alignments is called *the Pareto optimal alignment set*.

Under a multiobjective formulation, no weights are needed to be set up. Moreover, according to a classical result in the multiobjective optimization field [5], this optimal set contains not only all of the optima of a weighted sum formulation, but also many other alignments that are not possible to find at all by the weighted sum approach. Each of these alignments can be seen as a potential explanation of the relationship between the sequences and may be of interest for the practitioner for a more in-depth analysis. In fact, several other problems in bioinformatics have been already reformulated from a multiobjective point of view [6].

A multiobjective approach to pairwise sequence alignment has been explored by several researchers, both from a problem formulation and algorithmic point of view [7-11]. Recently, it has been applied to the construction of phylogenetic trees, which has shown to provide complementary information to that obtained by common methods [9].

## Implementations

MOSAL is a software tool that results from the problem formulation given in [9] with the aim of providing an open-source implementation and an on-line application where this implementation can be tested. The web-server is available at http://mosal.dei.uc.pt and physically located at the Department of Informatics Engineering, University of Coimbra, and is one of the outcomes of a national funded research project on multiobjective sequence alignment.

### Code

The code is written in C and provided under a GNU General Public License. A makefile is available for compilation under GNU/Linux. The implementation can be setup for several multiobjective score functions as described in [9]: maximization of the number of matches or substitution score and minimization of gaps or indels.

Speed-up techniques described in [9] are also implemented and can be parameterized, in particular, the maximum size of the lower bound set for the pruning technique. This parameter should be defined with some care; if too small, the pruning has a reduced effect, and if too large, a excessive number of comparisons may reduce the advantage of pruning in terms of CPU-time. For most of the benchmarks tested, a value of 10 seems to be the most appropriate [9].

The command line options available are described in Table 1. The implementation outputs the Pareto optimal set of alignments and the corresponding score function values by default.

**Table 1:** Command line options

**Option**	**Explanation**
F1	Path to the 1st sequence file (FASTA)
F2	Path to the 2nd sequence file (FASTA)
i|g	Use indels or gaps
dp | dpp -b=N	Do not use or use pruning technique.
	If yes, specify the size of lower bound (N)
-ss=F	Use substitution score instead of matches
	(F is the path to the matrix file)
–no-traceback	Output only the scores without the alignments

### On-line application

The web-server provides also an on-line application, written in PHP, that is available for sequences up to 2000 symbols. Four steps are needed to produce the set of Pareto optimal alignments: 

•Insertion of each sequence in FASTA format in a text box. The user can choose either Protein or DNA type of sequence in a switch button.

•Choice of the score function with switch buttons. The user can choose either matches or substitution score for the first score function component and either indels or gaps for the second score function component. If substitution score is chosen, the user can choose a substitution score matrix (PAM 100, 250 and BLOSUM 62, 75, 80, 85 if Protein option is chosen in the previous step) or can even provide one in a predefined text format.

•Choice of the sequence alignment options: with or without the alignments and with or without pruning technique. If pruning is chosen, the number of bounds must be provided (10 is given by default). The option without alignment provides only the score function values of the alignments.

•Submit to the server, with the option of sending an e-mail to the user with the output files.

**Figure 1:**
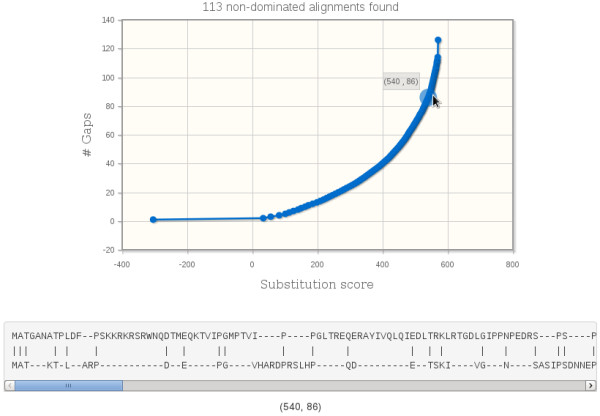
**Screen shot of the on-line application.** The figure illustrates the output of the on-line application for two sample sequences. The plot in the top shows the score function values as points for substitution score (bottom axis) and number of gaps (left axis). The alignment for a given score function value is given below.

Once the Pareto optimal alignment set is computed, the score function values are shown in an iterative plot; the user can zoom and choose a given point to see the corresponding alignment, see Figure 1. No information about the submissions is stored in the web-server. During the benchmark testing, the application was able to retrieve the output in less than 10 seconds for the largest sizes.

A visualization tool in the on-line application allows to visualize all the alignments and the corresponding score function values produced by the implementation or by the on-line application. The coloring scheme used in the Sequence Manipulation Suite (see http://www.bioinformatics.org/sms2/) is also applied here to allow the identification of potential regions of interest in the several alignments.

## Conclusions

MOSAL provides a set of tools for the practitioner to perform a more in-depth analysis on the relation between a pair of biological sequences. The multiobjective formulation that is explored by the framework provides further insight into the confidence of the alignments obtained by common methods; for instance, a large number of optimal scores suggests that a single alignment may be insufficient to understand the relation between the sequences and that further investigation is required. Moreover, the output can be used to construct phylogenetic trees as suggested in [9].

## Competing interests

The authors declare that they have no competing interests.

## Authors’ contributions

LP is the principal investigator of the project, PM implemented the application in the web-server, MA developed the problem formulation and algorithms, MP tested and suggested improvements in the framework. All authors read and approved the final manuscript.
